# *In Vivo* and *In Vitro* Toxicity Evaluation of Polyprenols Extracted from *Ginkgo biloba* L. Leaves

**DOI:** 10.3390/molecules201219839

**Published:** 2015-12-11

**Authors:** Cheng-Zhang Wang, Jiao-Jiao Yuan, Wen-Jun Li, Hong-Yu Zhang, Jian-Zhong Ye

**Affiliations:** 1 Institute of Chemical Industry of Forest Products, Chinese Academy of Forestry, Nanjing 210042, Jiangsu, China; yuanjj88lhs@163.com (J.-J.Y.); liwenjun611@163.com (W.-J.L.); chemicalzhy@163.com (H.-Y.Z.); yejianzhong1984@163.com (J.-Z.Y.); 2 Key and Open Laboratory on Forest Chemical Engineering, State Forestry Administration, Nanjing 210042, China; 3 Key Laboratory of Biomass Energy and Material, Institute of Chemical Industry of Forest Products, Nanjing 210042, China; 4 Institute of New Technology of Forestry, Chinese Academy of Forestry, Beijing 100091, China

**Keywords:** polyprenols, *Ginkgo biloba* leaves, toxicity, acute toxicity, subchronic toxicity

## Abstract

Polyprenols of *Ginkgo biloba* L. leaves (GBP) are a new type of lipid with 14–24 isoprenyl units, which in humans have strong bioactivity like the dolichols. A large amount of work showed that GBP had good antibacterial activity and powerful protective effects against acute hepatic injury induced by carbon tetrachloride and alcohol, as well as antitumor activity, but the safety of GBP was not considered. The current study was designed to evaluate the toxicity of these polyprenols. Acute toxicity in mice was observed for 14 days after GBP oral dosing with 5, 7.5, 10, 15 and 21.5 g/kg body weight (b. wt.) Further, an Ames toxicity assessment was carried out by plate incorporation assay on spontaneous revertant colonies of TA97, TA98, TA100 and TA102, with GBP doses designed as 8, 40, 200, 1000 and 5000 μg/dish, and subchronic toxicity was evaluated in rats for 91 days at GBP doses of 500, 1000 and 2000 mg/kg b. wt./day. The weight, food intake, hematological and biochemical indexes, the ratio of viscera/body weight, and histopathological examinations of tissue slices of organs were all investigated. The results showed that no animal behavior and appearance changes and mortality were seen during the observation period with 21.5 g/kg GBP dose in the acute toxicity test. Also, no mutagenicity effects were produced by GBP (mutation rate < 2) on the four standard *Salmonella* strains (*p* > 0.05) in the Ames toxicity test. Furthermore, the no observed adverse effect level (NOAEL) of GBP was 2000 mg/kg for 91 days feeding of rats in the subchronic toxicity tests. Results also showed the hematological and biochemical indexes as well as histopathological examination changed within a small range, and all clinical observation indexes were normal. No other distinct impacts on cumulative growth of body weight, food intake and food utilization rate were discovered with GBP. No significant difference was discovered for the rats’ organ weight and the ratio of viscera to body weight (*p* > 0.05). Reversible pathological changes in the histopathological examinations of tissue slices of organs were not observed. GBP could therefore be considered as a safe material with minor side effects.

## 1. Introduction

Plant polyprenols (PP), which exist widely in angiosperms, gymnosperms and fungi germs, *etc.* [1], are a type of isoprenoid lipid with different isoprene unit chain lengths, which display three structure types: the all *trans*-(*E*)-configuration of solanesol, the three *trans*-(*E*)-configuration of ficaprenol and the betulaprenol configuration [2]. Polyprenols are low molecular natural bioregulators that play a significant role in the cellular modulating process referred to as biosynthesis in plants. Polyprenol families can serve as chemotaxonomic markers for systematic families in botanic taxonomy [3], and could be considered as a “label” which grants the possibility to recognize infection in the innate immune system at the early stages and govern the acquired immunity [4]. PP and their phosphate esters are constituents of natural membranes in prokaryotic and eukaryotic cells, and can be transformed into dolichol and dolichyl phosphate by enzyme catalysis, forming the dolichol phosphate (DHP) cycle which mainly takes part in the biosynthesis of glycoproteins of all sorts of biomembrane structures *in vivo* as key glycoprotein carriers [5].

Previous studies have reported that polyprenols (*n* = 10–24) from pine needles had good biological functions [4,6,7]. *Pinus* needle polyprenols (PNP) were not toxic and very safe, acted particularly as antiviral agents, and clinical experiments showed that PNP had 60%~90% good inhibition and therapeutic effect on canine enteritis, canine infectious hepatitis, murine hepatitis, cat infectious gastroenteritis, cat infectious enteritis and peritonitis, bovine leukemia, rabies, and distemper virus [8].

Polyprenols of *Ginkgo biloba* leaves (GBP)*,* novel natural active lipids, were discovered in *G. bilabo* leaves. At present, the bioactive compounds of flavonoids and terpene lactones from *G. bilabo* leaves have already been widely used in the clinical treatment and prevention of cardiovascular and cerebrovascular diseases [9], however, the study of lipids of *G. biloba* leaves has rarely been reported in the literature, particularly GBP, and no GBP products have been developed.

**Figure 1:**
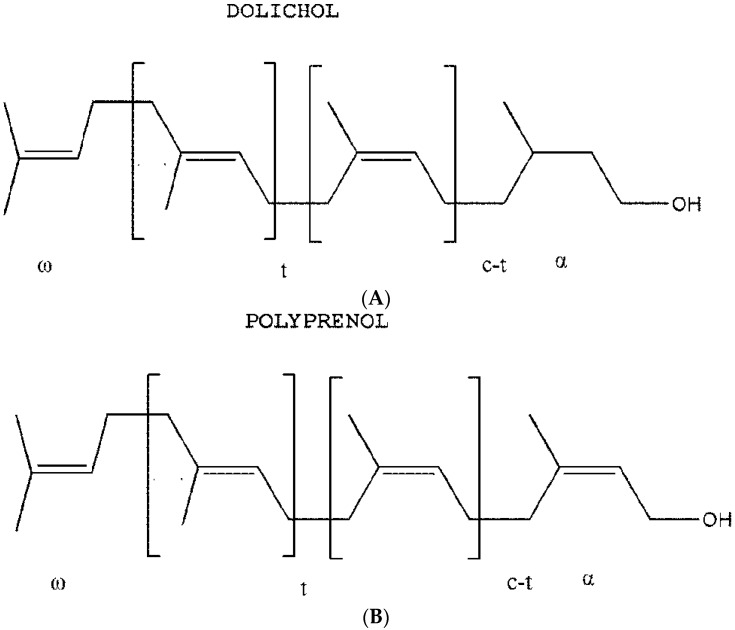
Chemical structure of dolichols and polyprenols of *Ginkgo biloba* leaves. (**A**) Dolichols; (**B**) Polyprenols of *Ginkgo biloba* L. (GBP).

GBP, generally consisting of 15 to 24 unsaturated isoprene units, belong to the betulaprenol type of ω-(*trans*)_2_-(*cis*)n-(cisα) type with an *E*,*E*-farnesyl residue at the ω-end of the prenyl chain and terminated by an isoprene unit bearing a primary hydroxyl group, and have similar structures and bioactivity as dolichols in human and mammalian organs where ω and α are terminal units (Figure 1) [10].

GBP have similar structure and pharmacology as the *Pinus* needle polyprenols (PNP), which have been developed as health foods, cosmetics and clinical medicines in Russia, Latvia, Poland and Australia, *i.e.*, Ropren and Bioactive-R in Russia and Austria. However, there has been no similar GBP preparation development up to now. In fact, a large body of work has showed that GBP has good antibacterial activity against *Salmonella enterica*, *Staphylocococus aureus*, *Aspergillus niger*, *Escherichia coli* and *Bacillus subtilis* [11], and it exerts a powerful protective effect on acute hepatic injury induced by carbon tetrachloride and alcohol [12,13] as well as antitumor activity [14,15], *etc.*, but in the current market there are no health foods or medicines based on GBP yet. The key reason is the lack of the safety evaluation of GBP.

In order to promote the comprehensive development and utilization of *G. biloba* leaves*.*, this research proposed to evaluate the acute toxicity and Ames toxicity of mice, then the subchronic toxicity of rats consecutively administered with GBP on a daily basis for 91 days, and changes such as haematological profile, body weight, ratio of selected viscera to body weight, biochemical indexes and histopathological examination, *etc.* were analyzed. This is a very important investigation to promote the development and the utilization of *G. biloba* and the polyprenol lipids from its leaves.

## 2. Results and Discussion

### 2.1. Determination of GBP

**Figure 2:**
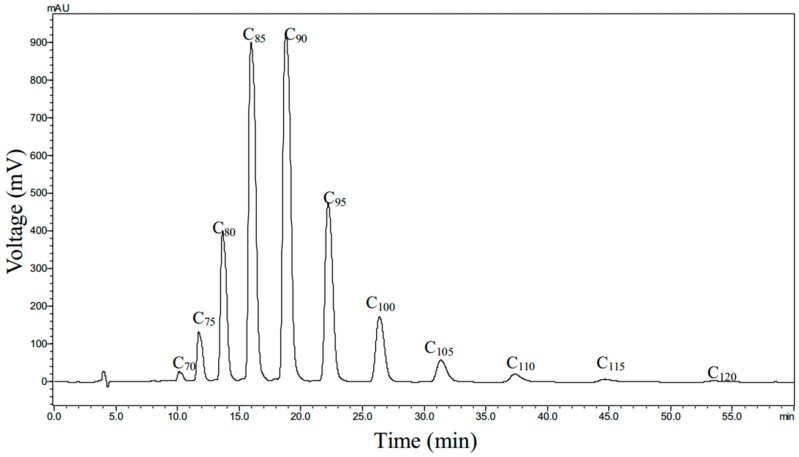
HPLC of GBP.

The yield of purified GBP was 0.35% of dried *G.* *biloba* leaves. Ethyl acetate/petroleum ether (1/9, *v*/*v*) mixture was used as developing solvent for TLC and the spots were detected by iodine for color development. The R_f_ value of GBP was 0.35. The polyprenols from *G.* *biloba* leaves were analysed by HPLC methods. The curve of standard polyprenols from *G. biloba* leaves (C_75_–C_105_), which displayed a better linear relation between injection weight (4.6~23.3 μg) and peak area, was expressed by the regression equation y = 1.5751x + 1.1546, R^2^ = 0.9991. The retention times of GBP with different numbers of isoprenyl units were 10.127 min (C_70_), 11.724 min (C_75_), 13.651 min (C_80_), 15.966 min (C_85_), 18.782 min (C_90_), 22.216 min (C_95_), 26.393 min (C_100_), 31.361 min (C_105_), 37.368 min (C_110_), 44.663 min (C_115_) and 53.519 min (C_120_), respectively. The purity of GBP, calculated according to the regression equation curve of the standard polyprenols, was up to 91.3%, and polyprenols were dominant in Figure 2.

### 2.2. The Acute Toxicity Accessment

In the acute toxicity test, the GBP dose was set at 5, 7.5, 10, 15 and 21.5 g/kg b. wt., and mixed with the salad oil to 20 mL. The mice took orally 20 mL/kg b. wt. GBP solution two times in 4 h intervals, and then were observed during 14 days. No animal behavior or appearance changes and mortality were noted during the observation period. A half lethal dose (LD_50_) could not be obtained, because GBP caused temporary gastrointestinal disorders within 24 h when the oral gavage dose was greater than 21.5 g/kg. The maximum drug dosage exceeded 21.5 g/kg, which was 25 times higher than the expected clinical daily dosage of human (60 kg. b. wt.). Moreover, compared with the weight change of the control group (19.6g), male mice changed from (20.5 ± 1.3) g to (34.1 ± 1.9) g, and female mice changed from (19.4 ± 0.7) g to (28.6 ± 0.7) g. The three groups all had a similar increasing tendency. The main organs (heart, liver, spleen, kidney) had no swelling, bleeding and necrosis symptoms during the 14 days observation period. No difference at a statistical significance *p* > 0.05 was observed between the female and male groups in body weight growth and organ to body weight ratios in Table 1 and Table 2. Biochemical indexes of RBC, WBC, HGB, ALT and AST had normal ranges compared with the control, and there was clearly no statistical difference in mice (*p* > 0.05) in Table 3. Therefore, the acute oral toxicity assay showed GBP did not have any obvious toxicity.

**Table 1:** Effects of GBP on body weight in mice (x ± s, *n* = 10).

Groups	Initial b. wt. (g)	Final b. wt. (g)	Survivor Number (*n*)	Mortality (%)	Maximum Drug Dosage (g/kg)
Control (50%♂ and 50%♀)	19.6 ± 0.4	31.8 ± 0.2	10	0	> 21.5
Female (♀)	19.4 ± 0.7	28.6 ± 0.7	10	0	> 21.5
Male (♂)	20.5 ± 1.3	34.1 ± 1.9	10	0	> 21.5

**Table 2:** Effects of GBP on ratio of organ to body weight in mice (x ± s, *n* = 10).

Groups	Liver/Body	Kidneys/Body	Heart/Body	Speen/Body
Control (50%♂ and 50%♀)	4.89 ± 0.04	0.88 ± 0.02	0.48 ± 0.02	0.31 ± 0.01
Female (♀)	5.03 ± 0.07	0.87 ± 0.03	0.49 ± 0.02	0.32 ± 0.02
Male (♂)	4.92 ± 0.02	0.85 ± 0.01	0.47 ± 0.01	0.29 ± 0.01

**Table 3:** Effect of GBP on haematological indexes in mice.

Groups	RBC (10^12^/L)	WBC (10^9^/L)	HGB (g/L)	ALT (IU/L)	AST (IU/L)
Control (50%♂ and 50%♀)	9.67 ± 0.16	6.22 ± 1.09	158.27 ± 14.31	34.87 ± 1.24	176.72 ± 14.22
Female (♀)	11.01 ± 0.35	5.45 ± 1.15	169.15 ± 2.47	35.25 ± 2.12	177.45 ± 12.26
Male (♂)	11.01 ± 0.35	5.45 ± 1.15	169.15 ± 2.47	35.25 ± 2.12	177.45 ± 12.26

### 2.3. Effects of GBP on the Mutagenicity of Standard Strains in Vitro

Ames toxicity results are shown in Table 4. The mean spontaneous revertant colonies of four strains were within the normal allowable range within or without S9 conditions. The positive control drugs Dexon (50 μg/dish) and 2-AF (10 μg/dish) showed significant differences (** *p* < 0.01), and produced two-fold more mean revertant colonies than the negative group on strains TA98, TA97a, TA100 and TA102, while each GBP test group clearly didn’t showed a statistical difference (*p* > 0.05) in doses of 8, 40, 200, 1000 and 5000 μg/dish., which resulted in lower mean revertant colonies and had no dose–response relationship. The test was repeated three times, and the results indicated that no mutagenicity effects (mutation rate < 2) were produced in the four standard *Salmonella* strains by GBP under the experimental conditions.

**Table 4:** Effects of GBP on revertant colonies of the standard strains *in vitro* (x ± s, *n* = 3).

Groups	Dosages	TA98		TA97a		TA100		TA102	
		−S9	+S9	−S9	+S9	−S9	+S9	−S9	+S9
First time									
Spontaneous revertant	0.1 mL/dish	31 ± 1	-	134 ± 14	-	153 ± 17	-	262 ± 20	-
Double-distilled water	0.1 mL/dish	31 ± 3	34 ± 3	144 ± 9	159 ± 18	163 ± 20	166 ± 25	254 ± 22	261 ± 25
DMSO	0.1 mL/dish	32 ± 3	31 ± 2	137 ± 13	147 ± 17	158 ± 22	163 ± 22	256 ± 29	264 ± 27
GBP	8 μg/dish	33 ± 2	33 ± 2	150 ± 15	157 ± 16	160 ± 21	158 ± 24	257 ± 26	266 ± 29
GBP	40 μg/dish	32 ± 3	32 ± 2	140 ± 12	151 ± 17	166 ± 22	161 ± 20	268 ± 22	265 ± 25
GBP	200 μg/dish	33 ± 1	33 ± 4	143 ± 12	149 ± 16	152 ± 21	166 ± 22	261 ± 25	257 ± 28
GBP	1000 μg/dish	31 ± 2	34 ± 3	138 ± 14	141 ± 13	160 ± 20	159 ± 21	253 ± 23	268 ± 26
GBP	5000 μg/dish	30 ± 1	33 ± 2	135 ± 10	150 ± 15	156 ± 19	163 ± 21	247 ± 24	257 ± 27
Second time									
Spontaneous revertant	0.1 mL/dish	31 ± 2	-	142 ± 12	-	153 ± 19	-	257 ± 28	-
Double-distilled water	0.1 mL/dish	31 ± 3	33 ± 2	131 ± 15	143 ± 16	150 ± 20	161 ± 21	255 ± 24	259 ± 26
DMSO	0.1 mL/dish	32 ± 2	34 ± 3	138 ± 13	151 ± 17	157 ± 19	162 ± 23	248 ± 25	269 ± 27
GBP	8 μg/dish	31 ± 1	32 ± 2	129 ± 19	149 ± 17	159 ± 21	165 ± 22	252 ± 25	270 ± 27
GBP	40 μg/dish	33 ± 2	34 ± 3	145 ± 10	153 ± 16	160 ± 18	170 ± 21	265 ± 22	259 ± 29
GBP	200 μg/dish	30 ± 2	33 ± 2	131 ± 12	141 ± 19	152 ± 20	161 ± 24	246 ± 26	245 ± 28
GBP	1000 μg/dish	32 ± 2	32 ± 2	148 ± 14	153 ± 15	156 ± 18	165 ± 20	252 ± 25	265 ± 32
GBP	5000 μg/dish	31 ± 1	32 ± 1	135 ± 10	146 ± 16	161 ± 19	159 ± 25	258 ± 28	269 ± 31
Dexon	50 μg/dish	1145 ± 115 **		2355 ± 263 **		1311 ± 171 **		1304 ± 159 **	
2-AF	10 μg/dish	2239 ± 255 **		1182 ± 145 **		2108 ± 232 **		-	
1,8-DHA	50 μg/dish	-		-		-		751 ± 100 *	

* Compared with negative control, the difference was statistically significant, *p* < 0.05; ** *p* < 0.01; “-” means no effect on revertant colonies of the standard strain.

### 2.4. The Subchronic Toxicity Accessment

#### 2.4.1. Animal Clinical Observation

**Figure 3:**
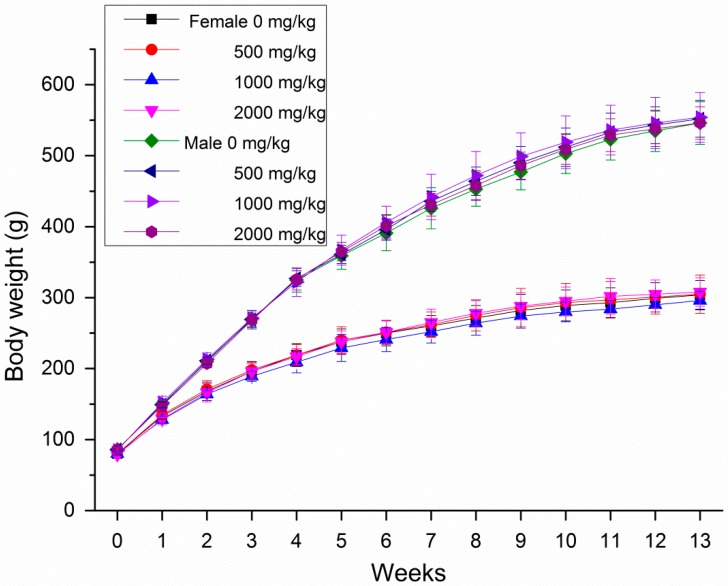
Body weight of female and male rats treated with GBP per week for 91 days.

**Figure 4:**
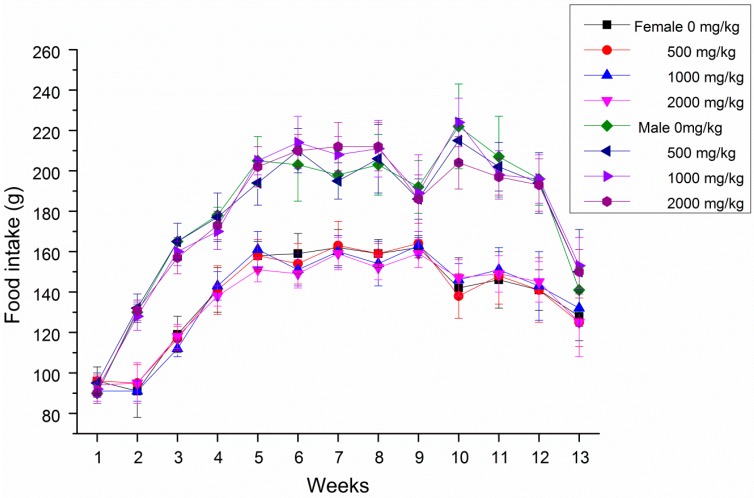
Food intake of female and male rats treated with GBP per week for 91 day.

**Figure 5:**
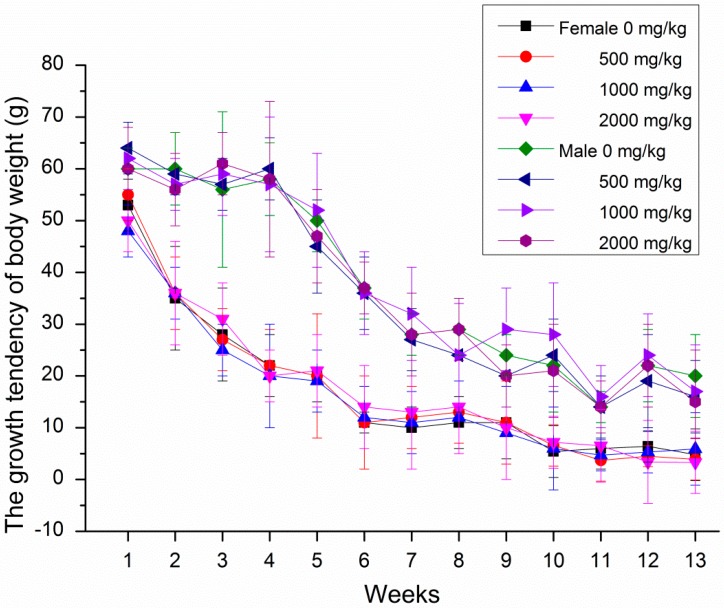
Growth tendency of body weight of rats per week treated with GBP for 91 days.

**Figure 6:**
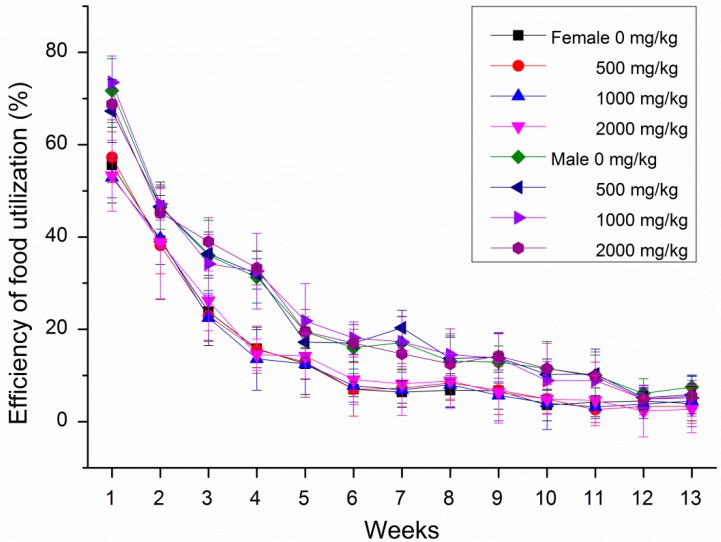
Efficiency of food utilization per week of rats treated with GBP for 91 days.

During the subchronic toxicity tests, no obvious symptoms of poisoning and death were observed in all rats after oral gavage of GBP doses of 500, 1000 and 2000 mg/kg b. wt. groups. Male rats increased the body weight and food intake more significantly than female rats treated with GBP per week during 13 weeks in Figure 3 and Figure 4. However, the body weight growth and food utilization tendency of rats per week for 91 days were similar (Figure 5 and Figure 6). Compared with the controls, no distinct impacts on cumulative body weight growth, food intake and food utilization rate of rats receiving different level of GBP for 91 days were observed (Table 5). All observation indexes and organs were normal and no mortality, ophthalmic abnormalities or treatment-related clinical signs were found during the study.

**Table 5:** Effects of GBP on body weight, food intake and food utilization rate after rats were treated for 91 days (x ± s, *n* = 10).

Sex	GBP Dose (mg/kg)	Initial b. wt. (g)	Final b. wt. (g)	Cumulative Body Mass Growth (g)	Total Food Intake (g)	Total Food Utilization (%)
Female	0	80 ± 4	304 ± 20	224 ± 21	1803 ± 42	12.4 ± 1.1
	500	80 ± 4	305 ± 27	225 ± 27	1796 ± 48	12.6 ± 1.7
	1000	80 ± 4	296 ± 13	215 ± 14	1797 ± 27	12.0 ± 1.1
	2000	79 ± 3	308 ± 20	229 ± 19	1781 ± 36	12.9 ± 1.1
Male	0	86 ± 4	546 ± 30	460 ± 30	2330 ± 106	19.8 ± 1.3
	500	86 ± 4	552 ± 26	466 ± 26	2322 ± 42	20.1 ± 0.9
	1000	85 ± 4	554 ± 35	469 ± 35	2348 ± 87	20.0 ± 1.6
	2000	86 ± 4	546 ± 23	460 ± 22	2310 ± 52	19.9 ± 1.1

#### 2.4.2. Effects of GBP on Biochemical Indexes

The hematological indexes of WBC, RBC and HB were evaluated at the midterm and final test phases (Table 6). Male rats had obviously higher blood cell counts (CBC) than female rat groups, however, a positive relation between GBP dose and cell counts was not observed. Particularly, RBC and HB were both more in final phase than in midterm, and it meant GBP were an important factor to impact the CBC index during the days of feeding. However, compared with the blank control groups, CBC of WBC, RBC and HB were all in the normal range in the different test phases, and there were no significant differences (*p* > 0.05). WBC classification count was also evaluated (Table 7). Compared with the blank control groups, BA produced a definite difference at the high dose of GBP 2000 mg/kg in final test male rats (* *p* < 0.05). Other dose groups did not cause a significant difference (*p* > 0.05) in WBC classification count of NE, LY, MO and EO, and they were all in the normal range during the different test phases.

GBP caused obvious changes on biochemical indexes of serum after final test on rats fed GBP (Table 8). Compared with the blank control groups, GBP reduced obviously female rats serum AST at middle dose of 1000 mg/kg (* *p* < 0.05) and GLU at middle dose or high dose of 2000 mg/kg (* *p* < 0.01), which implied GBP decreased blood glucose. However, statistical difference was not observed from other serum biochemical indexes change. For male rats, GBP could raise definitely ALT at middle and high dose (* *p* < 0.01), and reduce obviously BUN and CHO at high dose of 2000 mg/kg (* *p* < 0.05), and CR and TG at middle dose or high dose (* *p* < 0.01), which indicated GBP could reduce blood lipids. Besides, the other biochemical indexes of male and female mice were all in the normal range and not significantly different compared with the blank control groups (*p* > 0.05). 

On the whole, the changes of the above indexes in a small range could not prove GBP had any adverse biological significance. After administration of different doses of GBP for 91 days, the weight of organs, *i.e.*, liver, kidney and spleen of male rats increased more obviously than that of female rats, however female rats displayed higher ratios of viscera to body weight, *i.e.*, liver to body weight, than male rats. Compared with the control group values, no significant difference was discovered for rats’ organ weight and the ratio of viscera to body weight (*p* > 0.05) (Table 9). Histopathological examinations of tissue slices of liver, kidney, spleen, stomach, intestine, testes, and ovary showed no reversible pathological changes (Table 10).

**Table 6:** Effect of GBP on hematological cell counts (CBC) (x ± s, *n* = 10).

Sex	Doses (mg/kg)	Midterm Test (Day 46)			Final Test (Day 91)		
		WBC (10^9^/L)	RBC (10^12^/L)	HB (g/L)	WBC (10^9^/L)	RBC (10^12^/L)	HB (g/L)
Female	0	10.1 ± 3.6	5.93 ± 0.58	113 ± 10	9.4 ± 1.3	6.63 ± 0.31	140 ± 5
	500	11.0 ± 4.7	6.23 ± 0.52	120 ± 10	10.0 ± 1.0	6.72 ± 0.40	143 ± 5
	1000	10.1 ± 3.2	5.46 ± 0.48	105 ± 9	8.8 ± 1.4	6.79 ± 0.43	140 ± 10
	2000	11.0 ± 3.3	5.77 ± 0.59	112 ± 11	10.5 ± 2.4	6.46 ± 0.38	142 ± 6
Male	0	17.0 ± 4.2	5.92 ± 0.53	117 ± 9	12.8 ± 1.4	7.19 ± 0.71	146 ± 14
	500	16.0 ± 3.6	6.24 ± 0.20	123 ± 6	13.1 ± 1.7	7.32 ± 0.44	150 ± 9
	1000	18.1 ± 3.1	6.28 ± 0.35	121 ± 5	13.4 ± 3.2	7.04 ± 0.62	144 ± 9
	2000	16.1 ± 4.9	6.13 ± 0.37	118 ± 6	12.5 ± 1.8	6.18 ± 0.28	140 ± 6

**Table 7:** Effect of GBP on WBC classification count (CBC) (x ± s, *n* = 10).

Sex	Doses (mg/kg)	Final Test (Day 91)					Midterm Test (Day 46)				
		NE (%)	LY (%)	MO (%)	EO (%)	BA (%)	NE (%)	LY (%)	MO (%)	EO (%)	BA (%)
Female	0	30.7 ± 3.5	67.1 ± 3.4	1.37 ± 0.57	0.5 ± 0.3	0.37 ± 0.19	19.1 ± 5.1	77.0 ± 6.5	0.61 ± 0.44	2.7 ± 1.6	0.62 ± 0.53
	500	31.7 ± 5.8	65.8 ± 5.6	1.47 ± 0.59	0.5 ± 0.4	0.46 ± 0.27	19.8 ± 10.1	76.2 ± 10.7	0.41 ± 0.26	3.2 ± 3.6	0.41 ± 0.31
	1000	33.6 ± 4.9	63.9 ± 5.1	1.19 ± 0.34	0.7 ± 0.3	0.55 ± 0.25	23.4 ± 5.8	72.8 ± 6.6	0.65 ± 0.51	2.5 ± 0.9	0.60 ± 0.27
	2000	33.9 ± 6.4	63.1 ± 6.8	1.63 ± 0.81	0.6 ± 0.3	0.62 ± 0.26 *	25.8 ± 9.1	70.8 ± 8.9	0.50 ± 0.24	2.3 ± 0.9	0.59 ± 0.48
Male	0	29.1 ± 5.6	69.1 ± 5.2	0.79 ± 0.30	0.5 ± 0.4	0.49 ± 0.24	28.4 ± 8.1	68.7 ± 8.8	0.89 ± 0.26	1.7 ± 0.8	0.40 ± 0.33
	500	30.2 ± 6.0	68.2 ± 6.1	0.86 ± 0.31	0.4 ± 0.2	0.34 ± 0.18	34.6 ± 6.5	62.0 ± 5.8	0.80 ± 0.24	1.7 ± 0.7	0.67 ± 0.26
	1000	26.6 ± 6.0	71.8 ± 5.9	0.92 ± 0.34	0.4 ± 0.2	0.33 ± 0.13	33.3 ± 6.4	64.2 ± 6.3	0.92 ± 0.36	1.2 ± 0.4	0.48 ± 0.23
	2000	25.1 ± 5.4	73.2 ± 5.6	0.87 ± 0.37	0.4 ± 0.2	0.47 ± 0.35	33.5 ± 10.1	63.0 ± 9.8	1.12 ± 0.63	1.6 ± 0.6	0.60 ± 0.41

Note: one-way analysis of variance and pairwise comparison, compared with the blank control groups,* *p* < 0.05.

**Table 8:** Effects of GBP on serum biochemical indexes in rats (x ± s).

Sex	Doses (mg/kg)	AST (U/L)	ALT (U/L)	BUN (mmol/L)	CR (μmol/L)	CHO (mmol/L)	TG (mmol/L)	GLU (mmol/L)	TP (g/L)	ALB (g/L)
Female	0	158 ± 37	31 ± 8	5.66 ± 0.67	83.1 ± 5.5	1.83 ± 0.30	1.53 ± 0.40	6.90 ± 0.59	73.2 ± 9.2	40.5 ± 3.8
	500	133 ± 35	28 ± 8	5.54 ± 1.17	86.1 ± 6.7	2.06 ± 0.40	1.84 ± 0.48	6.61 ± 0.70	78.0 ± 5.8	44.0 ± 6.3
	1000	127 ± 21 *	28 ± 6	5.56 ± 0.49	86.9 ± 5.7	1.95 ± 0.47	1.35 ± 0.59	5.98 ± 0.82 **	78.9 ± 5.6	43.7 ± 5.7
	2000	132 ± 13	28 ± 4	5.62 ± 1.01	84.5 ± 4.5	2.10 ± 0.53	1.24 ± 0.50	5.81 ± 0.58 **	76.8 ± 5.7	43.3 ± 6.7
Male	0	144 ± 18	29 ± 6	5.91 ± 0.92	88.1 ± 3.8	1.95 ± 0.49	1.95 ± 0.53	5.88 ± 0.35	72.8 ± 6.1	41.6 ± 5.5
	500	152 ± 21	30 ± 4	5.72 ± 0.62	84.0 ± 4.9	1.66 ± 0.24	1.62 ± 0.40	6.60 ± 0.54	71.9 ± 4.0	39.4 ± 1.4
	1000	157 ± 20	35 ± 5 **	5.66 ± 1.33	81.6 ± 5.1 **	1.70 ± 0.30	1.59 ± 0.46 *	6.50 ± 0.94	70.7 ± 3.0	39.7 ± 1.4
	2000	145 ± 19	38 ± 5 **	4.82 ± 0.56 *	81.4 ± 4.5 **	1.58 ± 0.32 *	1.36 ± 0.36 **	6.16 ± 0.62	72.1 ± 5.2	39.2 ± 1.4

Note: one-way analysis of variance and pairwise comparison, compared with the blank control groups, * *p* < 0.05, ** *p* < 0.01.

**Table 9:** Effect of weight of rats’ organs (x ± s).

Sex	Doses (mg/kg)	Liver	Kidney	Spleen	Testes	Liver/Body (%)	Kidney/Body (%)	Spleen/Body (%)	Testes/Body (%)
Female	0	8.46 ± 0.57	1.86 ± 0.08	0.56 ± 0.07	-	2.80 ± 0.32	0.61 ± 0.04	0.18 ± 0.02	-
	500	8.26 ± 0.96	1.92 ± 0.19	0.59 ± 0.07	-	2.71 ± 0.26	0.63 ± 0.06	0.19 ± 0.02	-
	1000	8.19 ± 0.50	1.87 ± 0.11	0.52 ± 0.07	-	2.77 ± 0.14	0.63 ± 0.04	0.18 ± 0.02	-
	2000	8.46 ± 0.68	1.92 ± 0.11	0.51 ± 0.07	-	2.75 ± 0.20	0.62 ± 0.05	0.18 ± 0.02	-
Male	0	14.48 ± 1.12	3.39 ± 0.28	1.00 ± 0.13	3.64 ± 0.63	2.65 ± 0.15	0.62 ± 0.05	0.18 ± 0.02	0.67 ± 0.11
	500	14.10 ± 1.03	3.44 ± 0.26	0.99 ± 0.18	3.95 ± 0.36	2.56 ± 0.17	0.62 ± 0.02	0.18 ± 0.03	0.72 ± 0.06
	1000	14.60 ± 1.70	3.39 ± 0.27	0.95 ± 0.12	3.67 ± 0.35	2.63 ± 0.18	0.61 ± 0.05	0.17 ± 0.01	0.67 ± 0.08
	2000	13.83 ± 0.89	3.28 ± 0.24	0.93 ± 0.16	3.65 ± 0.19	2.54 ± 0.21	0.60 ± 0.06	0.17 ± 0.03	0.67 ± 0.04

Notes: “-” means no organ weight and the ratio of organ/body weight about female rats.

**Table 10:** Effect of GBP on histopathological examination of rats (numbers).

Organs	Morphological Change	Control Group		High-Dose Group	
		Female	Male	Female	Male
Liver	Liver cytoplasm vacuoles	1	0	0	0
	Mild hyperemia	2	1	2	2
	Inflammatory cell infiltration	0	0	0	2
Kidney	Mild hyperemia	3	0	0	1
	Inflammatory cell infiltration	0	0	1	0
Stomach	Mild hyperemia	0	1	0	0
	Inflammatory cell infiltration	1	0	0	2
Intestines	Mild hyperemia	1	1	1	0
	Local intestines expansion	0	0	1	0
Spleen	Mild billd stasis	1	0	0	2
	Necrosis	0	0	0	0
Ovary	Hyperemia	0	-	0	-
Testes	Sperm cells decreases	-	0	-	0

Notes: “-” means no histopathological examination about organs of rats.

## 3. Experimental Section

### 3.1. Materials

*Ginkgo biloba* leaves were collected from over 30 year-old trees in October 2013 at Nanjing Forestry University. Silica gel GF254 plates was self-made. Standard polyprenols from *G. biloba* leaves (C_75_–C_105_) were purchased from Larodan Chemiclals Co. (Malmö, Sweden). DMSO, Dexon, 1,8-dihydroxyanthraquinone (1,8-DHA) and 2-aminofluorene (2-AF) were purchased from Sigma Co. (St. Louis, MO, USA). Biochemical Kit reagents were purchased from Chinese Biological Technology Co. (Nanjing, China).

### 3.2. Preparation and HPLC analysis of GBP

Powdered *G. biloba* leaves (1 kg) were extracted with petroleum ether at room temperature for 48 h to get the crude lipid. The extracted lipid mixture was saponified at 70 °C for 1 h with 95% ethanol containing 15% (*w*/*v*) of NaOH and 0.5% of pyrogallol. The unsaponifiable matter was extracted four times with petroleum ether. The collected organic phases were evaporated and the residue dissolved in a solvent mixture (acetone-methanol = 85:15, *v*/*v*), then refrigerated for 4 h at −10 °C, to precipitate some impurities. The filtrate afforded crude GBP as a brown oil. Crude GBP was further purified by flash column chromatography (200 mesh silica gel, Φ 2.5 × 40 cm), using petroleum ether (400 mL) and 5% ethyl ether/petroleum ether (*v*/*v*, 300 mL) as eluents.

HPLC measurement was performed on Shimadzu SPD-20A (Shimadzu, Tokyo, Japan) at room temperature at 210 nm (DAD detector scanning from 190 to 800 nm) and a 2.5 μm Thermo BDS HYPERSIL C18 (15 × 4.6 mm) column, using an isopropanol–methanol (64:36, *v*/*v*) solvent mixture as the mobile phase at 0.5 mL/min for 60 min. The concentration of the GBP sample was 4.35 mg/mL and the injection volume was 5 μL. The retention times of different carbon chain length GBPs was 10.127 min (C_70_), 11.724 min (C_75_), 13.651 min (C_80_), 15.966 min (C_85_), 18.782 min (C_90_), 22.216 min (C_95_), 26.393 min (C_100_), 31.361 min (C_105_), 37.368 min (C_110_), 44.663 min (C_115_) and 53.519 min (C_120_), respectively. The absorption wavelength range of GBP was 190~232 nm in scanning 190~800 nm with DAD detector and the maximum absorption wavelength of GBP was 207 nm (C_70_), 207 nm (C_75_), 208 nm (C_80_), 210 nm (C_85_), 210 nm (C_90_), 208 nm (C_95_), 207 nm (C_100_), 207 nm (C_105_), 207 nm (C_110_), 207 nm (C_115_) and 206 nm (C_120_), respectively. TLC and HPLC were used to determine GBP had a purity of 93.5%.

### 3.3 Acute Toxicity Test

Forty healthy white mice (ICR, half male and half female, 18~22 g of body weight were supplied by Shanghai SLAC Laboratory Animal Co., Ltd., and were randomly assigned into four groups (two male groups and two female groups) in the toxicity experiment. GBP was given orally at the dose of 5, 7.5, 10, 15 and 21.5 g/kg b. wt., and mixed with the salad oil to 20 mL. The mice were fed at a dose of 20 mL/kg b. wt. each time, which was equivalent to 125 times the daily administration dosage of humans (50 kg). The mice were fasted for 12 h before oral gavage and water was freely available. The mice took orally 20 mL GBP solution, and repeated the same volume oral gavage in 4 h intervals. Then after 4 h, the mice could be fed freely, and were observed during 14 days. 

After the normal treatment, the mice were continuously observed for their body weight, food intake, activity and mental conditions within 14 days. With GBP maximum drug dosage of male and female group, change of mice blood indexes and the main organs (heart, liver, spleen, kidney) were observed after 14 d. The main organs were washed with physiological saline, filtered, weighted, and calculated each viscera index (the ratio of each viscera weight and weight).

### 3.4. Ames Toxicity Test

Strains TA97, TA98, TA100, TA102, and TA1535 were supplied by the Jiangsu Provincial Center for Disease Control and Prevention. The bacterial concentration of each strain was above 10^9^ cells/mL overnight culture. Hepatic microsomal enzymes (S9) was induced from rat liver homogenates by Aroclor1254, and the concentration of S9 mixture was 10%. 500 mg GBP were dissolved in 10 mL solution of DMSO, and centrifuged. The supernatant was diluted 5-fold with DMSO.

The Ames toxicity test was measured by a plate incorporation assay. GBP doses used in the experiment were 8, 40, 200, 1000 and 5000 μg/dish. Positive drugs were 50 μg/dish of Dexon, 10 μg/dish of 2-AF, and 50 μg/dish of 1,8-DHA. Solvent groups (double-distilled water and DMSO) and spontaneous reverse mutation group were set 0.1 mL/dish. The plates were cultured 48 h at 37 °C, and the average number of colonies was calculated. 

### 3.5. Subchronic Toxicity Test

#### 3.5.1. Animal Clinical Observation

Forty male and forty female SD rats (75~90 g body weight) were obtained from Shanghai SLAC Laboratory Animal Co., Ltd. The animals were randomly assigned into four male and female groups of 10 rats, respectively, and given sterilized commercial rat pellets and tap water was provided *ad libitum.* The doses used in the experiment were 0 (control group), 500 mg/kg b. wt./day, 1000 mg/kg b. wt./day, and 2000 mg/kg b. wt./day, which was equal to the 60, 120, 240 times the daily administration dosage of an adult human (60 kg), respectively. GBP samples of 0, 25 g, 50 g, and 100 g were weighed out to mix into the basic feed to reach 4000 g, respectively, and rats were fed with 80 g/kg b. wt./day. for 91 days. Clinical observations were recorded daily. Animals were observed twice daily for possible illness and mortality. Body weights were measured prior to GBP treatment and twice weekly during the treatment. Weekly and total food consumption, as well as weekly and total efficiency of food utilization for each animal were calculated at the end of the tests. Blood samples were obtained in the middle of the study (day 46) and at the end of the tests (day 91) for measurement of hematology and clinical chemistry.

#### 3.5.2. Biochemical Estimation

At the end of the test, all animals were euthanized for necropsy. The organ-to-body weight ratios were determined. The blood samples were collected to determine the haematology indices from eyeballs into tubes containing ethylenediaminetetraacetic acid (EDTA) anticoagulant. The levels of aspartic transaminase (AST), alanine aminotransferase (ALT), blood urea nitrogen (BUN), creatinine (CR), cholesterol (CHO), triglyceride (TG), blood glucose (GLU), total protein (TP), and albumin (ALB) were determined in the serum of the rats. An automated haematologic analyzer was used to analyze the haematologic parameters of white blood cells (WBC), red blood cells (RBC), haemoglobin (HGB), neutrophils (NE), lymphocytes (LY), monocytes (MO), eosinophils (EO) and basophils neutrophils (BA), *etc.* Moreover, selected organs were weighted and recorded, and histopathological examinations were investigated on tissue slices of liver, kidney, spleen, stomach, intestine, testis, and ovary.

### 3.6. Statistical Methods

The SPSS Statistical System (SPSS for Windows 11.0, SPSS Inc., Chicago, IL, USA) was used to analyze the chemical pathology, body weight, organ weight, food consumption, and clinical pathology data, followed by testing for variance homogeneity. Data were presented as the mean ± SD. Standard analysis of variance (ANOVA) was used for statistical evaluation of the data. Dunnett’s multipie comparisons were used to analyze the significance of differences between control and treated groups. All statistical tests were performed at the *p* < 0.05 level of significance.

## 4. Discussion and Conclusions

PNP, which shows no toxicity and safety, have been widely used as the additive in health foods, cosmetics and medicine for 20 years in Russia, Australia, Latvia and Poland, *i.e.*, “ROPREN” preparations had been developed in Russia, and “BIOACTIVER” has been developed by the Solagran Co. in Australia. The daily oral doses of PNP were 10–2000 mg/kg b. wt., and the LD_50_ exceeded 20 g/kg. The daily injection doses of PNP were 10–1000 mg/kg b. wt., and LD_50_ was greater than 1 g/kg. It is proved that there have no side effects causing mutagenesis, malformation and cancer in humans.

Few reports about GBP toxicity evaluation research exist. Polyprenols play important roles in improving cell membrane fluidity, stability and permeability, and enhancing membrane fusion [16] due to the similar molecular structure and bioactivity of GBP and PNP. The chronic toxicity of polyprenol soft capsules from *G.* *biloaba* leaves were studied [17]. It was considered that no obvious effect on most biochemical indexes was observable, but the triglyceride (TG) content showed a decreasing trend compared to blank control, which was due to the different content of polyprenols and drug administration time. However, the doses of polyprenol soft capsule were 833, 1667 and 2500 mg/kg, and the polyprenols content in the capsule was 4%, thus the real content of GBP was 100 mg/kg. Research on the toxicity of low molecular chitosan and polyprenols from *G.* *biloaba* leaves [18] and its influences on micronuclear rates and p53, Gadd45 protein expression of radiated mice [19] were reported. With low molecular chitosan and polyprenols, the no observed adverse effect level (NOAEL) was 2400 mg/kg for the low molecular chitosan and 40 mg/kg for polyprenols in a 30 days feeding test. It is not the only material to perform toxicity evaluations for GBP.

A toxicological evaluation of GBP was carried out in this study, and the results showed the maximum drug dosage of GBP was 21.5 mg/kg in mice, and no animal behavior and appearance changes or mortality were seen in the observation period. Meanwhile, the mean spontaneous revertant colonies of TA97, TA98, TA100 and TA102 strains were within the normal allowable range, and the NOAEL of GBP was 2000 mg/kg for 91 days feeding of rats. At the dose of 2000 mg/kg, no significant negative effects on animal behavior and appearance changes and mortality occurred. On the whole, the changes of hematological and biochemical indexes as well as histopathological examination changed within a small range, and all clinical treatment observation indexes were normal. Cumulative growth of body weight, food intake and food utilization rate were not affected distinctly by GBP. No significant difference was observed for rats’ organ weights and the ratio of viscera to body weight (*p* > 0.05). Reversible pathological changes in the histopathological examinations of tissue slice of organs were not observed, therefore, GBP could be considered as a safe material with minor side effects.

In summary, *G.* *biloba* leaves are rich in bioactive compounds such as flavonoids, terpene lactones, polyprenols, lipids, *etc.* Extract of *G. biloba* leaves (EGB761) has been used as a herbal raw material in Europe and America. This paper shows the GBP have virtually no toxicity, therefore it provides a theoretical basis for the use of GBP as a raw material of health foods and drugs, and should promote the comprehensive research and development of *G. biloba* leaves*.*
